# COLLABORATIVE AGING RESEARCH USING TECHNOLOGY: NEW PATHWAYS FORWARD

**DOI:** 10.1093/geroni/igz038.3065

**Published:** 2019-11-08

**Authors:** Jeffrey Kaye, Zachary Beattie, Nicole Sharma, Thomas Riley, Lisa Silbert, Lisa Barnes, Sarah Czaja, Hiroko Dodge

**Affiliations:** 1 Oregon Health & Science University, Portland, Oregon, United States; 2 Rush University, Chicago, Illinois, United States; 3 Weill Cornell Medical College, New York, New York, United States

## Abstract

A profusion of technologies and protocols have been developed to more effectively assess and deliver care to older adults with cognitive impairment, challenged health and declining function. These technologies take advantage of important developments in sensing and pervasive computing, wearable technologies, mobile and wireless communications, and “big data” analytics. Despite great promise challenges remain to realizing their full potential and achieving wider uptake and dissemination in research and practice. This presentation will review and provide an overview of major technologies, their integration, and their use-cases, as well as key challenges present in the current landscape. The presentation will highlight ongoing developments addressing these challenges with particular attention to the Collaborative Aging Research using Technology (CART) initiative supported by the NIH and VA, an initiative directed toward providing an open technology research platform to be used by diverse investigators across the U.S. to facilitate and improve aging research using technology.

